# Additional evidence for the role of chromosomal imbalances and SOX8, ZNRF3 and HHAT gene variants in early human testis development

**DOI:** 10.1186/s12958-022-01045-7

**Published:** 2023-01-11

**Authors:** Khouloud Rjiba, Soumaya Mougou-Zerelli, Imen hadj Hamida, Ghada Saad, Bochra Khadija, Afef Jelloul, Wafa Slimani, Yosra Hasni, Sarra Dimassi, Hela Ben khelifa, Amira Sallem, Molka Kammoun, Hamza Hadj Abdallah, Moez Gribaa, Joelle Bignon-Topalovic, Sami Chelly, Hédi Khairi, Mohamed Bibi, Maha Kacem, Ali Saad, Anu Bashamboo, Kenneth McElreavey

**Affiliations:** 1 Laboratory of Human Cytogenetics, Molecular Genetics and Biology of Human Reproduction, Farhat Hached University Teaching Hospital, Sousse, Tunisia; 2 Higher Institute of Biotechnology Monastir, University of Monastir, Monastir, Tunisia; 3 Unité de Services Communs en Génétique Humaine, Faculté de Médecine de Sousse, Université de Sousse, Sousse, Tunisia; 4 Human Developmental Genetics Unit, CNRS UMR 3738, Institut Pasteur, Paris, France; 5 Department of Endocrinology, Farhat Hached University Teaching Hospital, Sousse, Tunisia; 6 Laboratory of Human Cytogenetics and Biology of Reproduction, Fattouma Bourguiba University Teaching Hospital, Monastir, Tunisia; 7 Private Gynecologist Sousse, Sousse, Tunisia; 8 Department of Gynecology and Obstetrics, Farhat Hached University Teaching Hospital, Sousse, Tunisia

**Keywords:** Disorders of sex development (DSD), 46,XY DSD, Cytogenetic abnormalities, Whole exome sequencing(WES)

## Abstract

**Background:**

Forty-six ,XY Differences/Disorders of Sex Development (DSD) are characterized by a broad phenotypic spectrum ranging from typical female to male with undervirilized external genitalia, or more rarely testicular regression with a typical male phenotype. Despite progress in the genetic diagnosis of DSD, most 46,XY DSD cases remain idiopathic.

**Methods:**

To determine the genetic causes of 46,XY DSD, we studied 165 patients of Tunisian ancestry, who presented a wide range of DSD phenotypes. Karyotyping, candidate gene sequencing, and whole-exome sequencing (WES) were performed.

**Results:**

Cytogenetic abnormalities, including a high frequency of sex chromosomal anomalies (85.4%), explained the phenotype in 30.9% (51/165) of the cohort. Sanger sequencing of candidate genes identified a novel pathogenic variant in the *SRY* gene in a patient with 46,XY gonadal dysgenesis. An exome screen of a sub-group of 44 patients with 46,XY DSD revealed pathogenic or likely pathogenic variants in 38.6% (17/44) of patients.

**Conclusion:**

Rare or novel pathogenic variants were identified in the *AR, SRD5A2, ZNRF3, SOX8, SOX9* and *HHAT* genes. Overall our data indicate a genetic diagnosis rate of 41.2% (68/165) in the group of 46,XY DSD.

## Background

Differences/Disorders of Sex Development (DSDs) are defined as congenital conditions with a discrepancy between chromosomal, gonadal, and phenotypic sex [1]. They represent a major clinical concern that is most often present in newborns or adolescents [2]. The prevalence of DSD is often underestimated since the diagnosis can be relatively late, at puberty or during adulthood and, in some countries, sexual issues are still sensitive, resulting in a reluctance to seek clinical counselling [3]. This may explain why in Saudi Arabia and Egypt, the incidence of ambiguous genitalia is estimated to be 1:2,500 and 1: 3,000 of live births, respectively, whilst in European countries it is estimated at 1: 4,500–1: 5,500 of live births [4–7].The data could also reflect the high rate of consanguinity, especially in developing countries, where autosomal recessive forms of DSD are more prevalent [8]. Population isolates may also contribute to the presence of rare or novel variants with a limited geographic range [8].

Forty-six ,XY DSD can be due to chromosome abnormalities or genetic variants in the genes involved in the development or function of the male gonad as well as anomalies of downstream target tissues [9]. In most studies, the genetic cause is established in less than 50% of 46,XY DSD cases [1, 9, 10]. At a molecular level pathogenic variants in the *AR, NR5A1, SRD5A2, ZFPM2, HSD17B3* and *DHH* genes are the most frequent causes of 46,XY DSD [9, 10]. The aim of this study was to define the genetic etiology in a large cohort of 46,XY DSD patients from a North African population and compare these data to those observed in other populations. The cytogenetic analysis and molecular gene approaches resulted in a combined diagnosis yield of 41.2% (68/165) for this DSD subgroup. Cytogenetic analysis detected autosomal or sex chromosome anomalies in 30.9% of all cases, whereas WES identified rare or novel variants in the *AR, SRD5A2, ZNRF3, SOX8, SOX9* and *HHAT* genes (17/44 cases; 38.6%). These results emphasize the usefulness of both cytogenetic approaches as well as exome sequencing to make an accurate genetic diagnosis for a better genetic counseling and knowledge-based management of this group of patients.

## Patients and methods

### Cohort and study design

A total of 165 patients with DSD were referred for genetic consultation in the department of Cytogenetic, Molecular biology, and Biology of Human Reproduction, Teaching hospital Farhat Hached, Sousse, Tunisia over a period of 3 years (2018–2020). The local Ethics Board of the University Teaching Hospital Farhat Hached approved the present study (IRB00008931) and written consents were taken from adult probands or from the parents when the patient was under 18 years. The patients presented with a range of clinical DSD profiles and their ages ranged from birth to 35 years. They underwent a complete clinical examinations, including genital examination, family history and examination for the presence of somatic abnormalities. Imaging examination and hormonal evaluation were also carried out according to each case. Patients with suspected or confirmed congenital adrenal hyperplasia (CAH) were excluded from this study. All patients are from Tunisian ancestry.

### Genetic analysis

#### Cytogenetic studies

Reverse Heat Giemsa (RHG) banded karyotype was performed on metaphase chromosome preparations obtained from peripheral blood lymphocytes of both patients and parents according to standard protocol (450–550 band level). A minimum of 20 R-banded metaphase chromosomes were analyzed using Cytovision® Karyotyping software version 4.0. Karyotypes were classified according to the International System of Human Cytogenetic Nomenclature (ISCN 2020) [11]. Fluorescent in situ Hybridization (FISH) was carried out on metaphase chromosomes of the patients according to the standard protocol, using commercial probes. Array Comparative genomic hybridization (aCGH) 4 × 44 K micro-arrays was performed using the Agilent platform according to the manufacturer’s instructions (Feature Extraction 9.1, CGH Analytics 4.5, Santa Clara, California, United States). An abnormal ratio greater than + 0.58 or lower than − 0.75 was considered as an alteration. An *in silico* analysis of the unbalanced regions was executed using UCSC Genome Browser (https://genome.ucsc.edu/), the Database of Chromosome Imbalance and Phenotype in Humans using Ensemble Resources (DECIPHER: https://decipher.sanger.ac.uk/), the Database of Genomic Variants (DGV: http://dgv.tcag.ca/dgv/app/home) and the Online Mendelian Inheritance in Man database (OMIM: https://omim.org/).

#### Sanger sequencing

Genomic DNA was extracted from the peripheral blood of the patient using the FlexiGene DNA Kit (Qiagen, Hilden, Germany). Direct Sanger sequencing was performed using the Big Dye Terminator V3.1 Cycle Sequencing, on the ABI 3730XL sequencer (Applied Biosystems, Foster City, CA, USA). Sequencing data were analyzed by SeqScape 2.0 software (Applied Biosystems).

#### Whole exome sequencing

The WES approach was performed on DNA from 44 XY individuals who had a complete clinical investigation including examination of genitalia, hormonal screens and, where possible, gonad histology. All of these patients presented with a broad spectrum of 46,XY DSD phenotypes for which the underlying cause is unknown.

Exonic and adjacent intronic sequences were enriched from genomic DNA using Agilent SureSelect Human All Exon V4, and paired-end sequencing was done with the TruSeq v3 chemistry on Illumina HiSeq2000 platform. Based on the manufacturer's proprietary software, reads were mapped using the Burrows-Wheeler Aligner. Single nucleotide variants (SNV) and small insertions or deletions (Indels) were generated with GATK 1.6 version. BAM files were also carried out using SAMtools version 0.1.18. GATK. Unified Genotyper software was used for calling single nucleotide polymorphism (SNP) and Indels variants for each patient.

The annotated VCF files were then formatted to be used as a Microsoft Excel spreadsheet software and a selection of variants according to well-defined criteria (degree of pathogenicity, type of variant, frequency of the variant in all populations, including sub population) was performed. Synonymous, intronic and non-coding RNA variants were removed. Missense, nonsense, insertion/deletion and splice-site variants that were homozygous with a Minor Allele Frequency (MAF) of > 0.01 were excluded and heterozygous variants with a MAF of > 0.001 according to the GnomAD database (https://gnomad.broadinstitute.org/) were also excluded.

According to the clinical data of each patient, the analysis of variants was performed through a range of web-based bioinformatics tools. The variant Effect Predictor(VEP) bioinformatics tool on the Ensembl website (http://www.ensembl.org/homosapiens/userdata/uploadvariations), gnomAD(https://gnomad.broadinstitute.org/),ClinVar (https://www.ncbi.nlm.nih.gov/clinvar/) and Database of Genomic Variants (http://dgv.tcag.ca/dgv/app/home) were used to annotated the novel variants.

The possible impact on protein structure and function was evaluated to determine the pathogenicity of the variants based on individual scores made by Sorting Intolerant from Tolerant (SIFT),Polymorphism phenotyping V2( PolyPhen2) and Rare Exome Variant Ensemble Learner (REVEL; [12]) tools.

The Clustal Omega tool (https://www.ebi.ac.uk/Tools/msa/clustalo/) was used to generate alignments between three or more protein sequences. The Hope tool was used to analyze the structural effects of a point variation in a protein sequence [13].

Clinical significance was established according to the 2015 American College of Medical Genetics and Genomics and Association for Molecular Pathology (ACMG) in order to establish a better genotype–phenotype correlation. [14]. Potentially pathogenic variants were verified by Sanger sequencing.

The WES cohort of 46,XY DSD consisted of 19 individuals raised as females and 25 raised as males. Within this group, 17 cases were syndromic and 27 cases non-syndromic cases.

## Results

The most common feature at consultation was atypical external genitalia (67 patients) or typical male external genitalia with azoospermia (42 patients). A total of 30 patients presented with other congenital anomalies including intellectual deficiency, dysmorphic features, heart defects growth delay and cerebral anomalies. Primary amenorrhea and delayed puberty were reported in 18 and 8 cases respectively (Table 1). In this cohort, the patients were classified into three groups: Sex chromosome DSD, autosomal chromosomal abnormalities and 46,XY DSD (Table 1). 66% of the studied patients (110/165) were diagnosed as having 46,XY DSD of whom 23% were raised as females.

**Table 1:** Presentation of different categories of studied DSD Tunisian cohort

Classification	Diagnostic criteria	No
**Sex chromosome DSD (No = 47)**	Azoospermia	42
	Atypical genitalia & congenital anomalies	2
	Atypical genitalia	3
**Autosomal chromosome anomalies**	DSD & congenital anomalies	8
**46,XY DSD (No = 109)**	Atypical genitalia	64
	Primary amenorrhea	18
	Delayed puberty	8
	DSD & congenital anomalies	20
**Total number 165**		

Abbreviations: DSD: Disorders of sex development

### Cytogenetic results

**Fig. 1:**
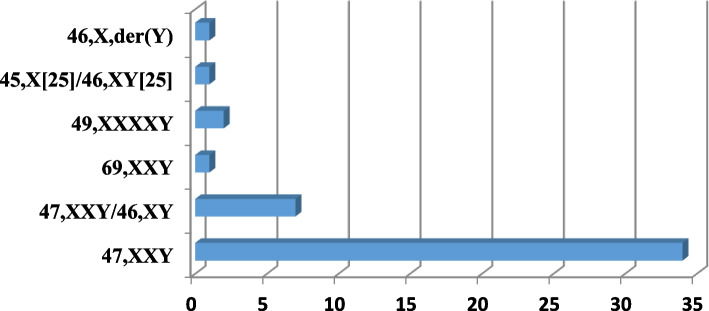
Distribution of patients with sex chromosome DSD according to their karyotype. 47,XXY was the most common observation

The proportions of different categories of DSD are shown in Table 1 and the distribution of patients with sex chromosome DSD in relation to their karyotype is illustrated in Fig. 1.

Sex chromosome anomalies were detected in 48/165 patients (29.1%) and autosomal chromosome abnormalities were detected in three individuals (1.8%). Klinefelter syndrome (KS) was the most prevalent chromosome sex abnormality in (87.2%) and genetic cause of azoospermia (83.3%) in males.

aCGH was performed on twenty patients based on their clinical presentation, suggesting a contiguous gene syndrome or an a priori assumption of the involvement of rearrangements affecting known gonadal genes or their regulatory sequences with various other extragonadal malformations. In 8 patients, anomalies were observed (Table 2).

**Table 2:** Clinical and cytogenetic findings in patients with DSD explored with aCGH (4 × 44 K)

Patient		Patient 1	Patient 2	Patient 3	Patient 4	Patient 5	Patient 6	Patient 7	Patient 8
Age		2 days	24 years	7 months	8 months and half	3 years	9 months	3 Years	1 year
Indication		Dysmorphic features	Primary amenorrhea	Growth delay	Growth delay	Dysmorphic features	polymalformative syndrome	Abnormal external genitalia	polymalformative syndrome
Consanguinity		No	Yes	Yes	No	No	No	yes	No
Sex of rearing		M	F	F	M	M	M	M	M
congenital anomalies associated		Dysmorphic features Microcephaly Inter-atrial communication heat defect	Mental retardation	Hypotonia, Dysmorphic features, psychomotor delay Inter-atrial communication heat defect	Dysmorphic features Microcephaly Psychomotor delay	Dysmorphic features Microcephly	Dysmorphic features microcephaly	Macrocephaly Dysmorphic features	Microcephaly Dysmorphic features Inter-atrial communication heat defect Arterial hypertension
Genitalia	**External**	Micropenis, hypospadias, cryptorchidism	Micropenis, palpable right testis	Female external genitalia	micropenis	micropenis	Micropenis, crytorchidism	Micropenis cryptorchidism	Micropenis Hypospadias
	**Internal**	NA	Absence of ovaries and uterus	Absence of uterine or ovarian structure	NA	NA	NA	NA	NA
	**Gonads**	Testis	Testis	Gonadal agenesis	Testis in inguinal position	Testis in inguinal position	Testis in the abdomen	Testis in inguinal position	Small testis
Karyotype		46,XY,(del4)(p16.3)	46,XY	46,X,der(Y)	46,XY,r(9)	46,XY,r(9)	45,XY,rob(13;1)(q10;q10)	46,XY,der(12)[4]/46,XY[16]	46,XY
FISH (*SRY* gene)		46,XY,(del4)(p16.3).ish(Yp11.)(SRY × 1)	46,XY.ish(Yp11.3)(SRY × 1)	46,X,der(Y).ish(Yp11.3)(SRY × 1)	46,XY,r(9).ish(Yp11.3)(SRY × 1)	46,XY,r(9).ish(Yp11.3)(SRY × 1)	45,XY,rob(13;14).ish(Yp11.3)(SRY × 1)	46,XY,der(12)[4] /46,XY[16].ish(Yp11.3)(SRY × 1)	46,XY.ish(Yp11.3)(SRY × 1)
aCGH (4 × 44 K)		46,XY.arr[NCBI36]4p16.3(62,447_9,065,971) × 1 dn **(deletion size: 4.8 Mb**)	46,XY.arr8p23[ NCBI36](7,290,597_11,665,267) × 1 ( **deletion size:4 Mb**)	46,X,der(Y)t(X;Y)(p21.3;p11.3).arr[ NCBI36]Xp21.2(2,710,316_30,233,793)X2 dn (**duplication size:27.5 Mb**)	46,XY,r(9).arr[ NCBI36]9p24-pter(601,628_1,569,467) × 1 ( **deletion size:1 Mb**)	46,XY,r(9).arr[ NCBI36]9p22,2(601,628_7,786,728) X1 (**deletion size ~ 6 Mb**)	45,XY,rob(13;14).arr[ NCBI36]8p23.2(184,617_7,290,647) × 1, 8p21.2(12,627,630 _30,839,917) × 3 **deletion size ~ 7 Mb**) (**duplication size ~ 18 Mb)**	46,XY,der(12)[4] /46,XY[16].mos [arr NCBI36]12p(179,323_30,681,410) × 3 dn (**duplication size:30 Mb)**	46,XY.arr[ NCBI36]16p12.1(29,582,349_30,106,101) × 1 **(Deletion size: 1 Mb)**
FISH Specific loci Probes		46,XY,(del4)(p16.3).ish(4p16.3)(WHSC1 × 1)	Blue FISH:RP11-52B19 46,XY.ish(8p23) ( RP11-52B19 × 1	46,X,der(Y).ish(Xp21.2)(NR0B1 × 2) 46,X,der(Y).ish(Xp22.3)(KAL1 × 2) 46,X,der(Y).ish(WCPX × 1)(WCPY × 1)	46,XY,r(9).ish(subtel9p × 1)(subtet9q × 2)	46,XY,r(9).ish(subtel9p × 1)(subtet9q × 2)	NA	46,XY,der(12).ish(WCP12[4] /46,XY[16]	NA
Inheritance		De novo	NA	De novo	NA	De novo	De novo	De novo	NA
*Initial clinical diagnosis*		Wolf Hirshhorn Syndrome	46,XY GD	46,XY GD	Alfi syndrome	Alfi syndrome	46,XY DSD		
*Candidate gene in association with the DSD phenotype*		MSX1(candidate gene)	GATA4	NR0B1	DMRT1-3	DMRT1-3	NA	NA	NA

Abbreviations: NA: not available; M: male, F:female; dn: de novo, DSD: disorders of sex development; FISH, Fluorescence in situ hybridization; aCGH: array comparative genomic hybridization

These included five cases with intra-chromosomal deletions, two cases of intra-chromosomal duplications, and one case with an inversion duplication/deletion (invdupdel) chromosome imbalance (Table 2). Of these chromosomal anomalies, genes known to cause 46,XY DSD were identified for 4 patients (Table 2) including *DMRT1, GATA4* and *NR0B1*. FISH analysis confirmed the heterozygous deletion of the *GATA4* gene in patient 2 and a duplication of the *NR0B1* gene in patient 3. The patient 1 presented the Wolf–Hirschhorn syndrome (WHS) [OMIM#194190]. In addition to the typical WHS phenotype, he presented a hypospadias, micropenis and cryptorchidism. The 4p16.3 deletion presumably results in haploinsufficiency of the *MSX1* gene [OMIM#142983] whose absence might be indirectly responsible for the hypospadias phenotype as this gene contributes to the spatiotemporal regulation of GnRH transcription during development [15]. In three patients (patients 6–8), there was no obvious candidate gene located within the chromosomal anomaly. Clinical details and cytogenetic results are summarized in Table 2.

### Sequencing data

Exome sequencing was performed in a total of 44 patients with 46,XY DSD. Amongst them, 27 were non-syndromic and 17 presented with somatic anomalies. Of the 44 patients, a genetic cause was established in 17 cases (38.6%) of whom 13 presented non-syndromic DSD form and 4 with syndromic forms. Likely benign (LB) and variants of uncertain significance (VUS) were identified in 11/27 non-syndromic individuals (40.7%) and 4/17 (23.5%) of syndromic individuals. Pathogenic and likely pathogenic variants in the following genes: *AR* (n = 6), *SRD5A2* (n = 2), *LHCGR* (n = 1), *ZNRF3* (n = 1), *HHAT* (n = 1), *SOX8* (n = 1), *IER3IP 1*(n = 1), *SRY* (n = 1), *SOX9* (n = 1), *FLNA* (n = 1) and *PEX1* (n = 1). The Clinical and molecular findings are summarized in Table 3.

**Table 3:** Clinical phenotype, hormonal profil and details of potentially pathogenic variants identified by WES in a cohort of individuals with 46,XY DSD

nDSD case	Age/sex of rearing	FSH (UI/l)/ Normal range	LH (UI/l)/ Normal range	Testosterone (ng/ml)/ Normal range	Genitourinary				Somatic features	Gene/Variant/Zygosity/inheritance	MAF and population (gnomAD)/ Predicted effect on protein	Clinical significance: ACMG/ ClinVar/ GV, Ref
					**External genitalia**	**Internal genitalia**	**Gonad position**	**Gonad Histology**				
**DSD1**	17Y/F	8.92 (6.3–24)	8.81 (9.6–80)	4.06 (0.7–19)	Micropenis, absence of labia minora, no vagina	Absent MD, presence of seminal vesicles, hypotrophic testis	R: inguinal, L: inguinal	Testis/ NA	-	SRD5A2:NM_000348:c.G344A:p.G115D,Hom, parents	0.0001964 Latino-Admixed American/ SIFT: D (0.03), PP2: PrD (0.993), REVEL: NA	P/NA / P [Lavinia vija et al 2014, Laurent Maimoun 2011]
**DSD2**	28Y/F	NA	NA	NA	Micropenis, absence of labia minora and majora, vaginal aplasia	Absent MD, atrophic testes	R: inguinal, L: inguinal	Testis /Sertoli cells, no germ cells, Leydig cell hyperplasia	-	SRD5A2:NM_000348:c.A622C:P.T208P, Hom, parents	Novel/SIFT: SIFT: D(0.01), PP2: PrD (0.913), REVEL: NA	LP/ NA/ NA
**DSD3**	30 Y/F	7 (1.7–12)	15.5 (1.1–7)	NA	F	Absent uterus, hypoplasic gonads	NA	NA	-	AR:NM_000044:c.G2231A:p.G744E, Hemi, maternal	NA/ SIFT: D(0.00), PP2: PrD(1.00), REVEL: 0.935 (LDC)	P/ P/ NA
**DSD4**	8Mo/F	NA	0.8 (2–12)	< 0.1(0.2–0.8)	F, bilateral inguinal hernia	Absent MD, presence of testes in hernia sac	Hernia sac	Testis/Sertoli cells with seminiferous tubes, no germ cells	-	AR:NM_000044:c.G2231A:p.G744E, Hemi,NA	NA/ SIFT: D(0.00), PP2: PrD(1.00), REVEL: 0.935 (LDC)	P/ P/ NA
**DSD5**	4D/F	NA	NA	NA	Hypertrophy of the clitoris and labia majora	NA	NA	NA	-	AR:NM_000044:c.A1742G:p.K581R, Hemi, maternal	Novel/ SIFT: D (0.00), PP2: PrD (0.998), REVEL: NA	LP/ NA/ NA
**DSD6**	18Y/F	NA	NA	NA	F	Absent MD, presence of bilateral gonadal mass with epididymes	NA	Two atrophic testes, presence of Sertoli cells, no germ cells	-	AR:NM_000044:c.G1597T:p.G533*, Hemi, matneral	Novel/LOF	P/ NA/NA
**DSD7** **DSD8 (sibs)**	25Y/F	28.7 (1.1–7)	5.6(1.1–7)	NA	F, bilateral inguinal hernia	Vagina present, absent MD	NA	no residual gonad	-	AR:NM_000044:c.C1277A::p.S426*, Hemi, *De novo*	Novel/LOF	P/ NA/ NA
	15Y/F	7.38(1.7–12)	10.46(1.1–7)	0.23(0.1–0.9)	F	Absent MD, presence of gonadal mass	R:Inguinal,L: no residual gonad	NA	-			
**DSD9**	1Y and half/M	NA	NA	NA	Hypospadias, cryptorchidism, incomplete foreskin	NA	NA	NA	-	AR:NM_000044:c.T170A::p.L57Q, Hemi, maternal	Novel/ SIFT:D (0.001), PP2: unknown, REVEL: 0.150(B)	B/LB/NA
**DSD10**	14Y	39(1.7–12)	NA	NA	F	Absent gonads and uterus	No residual gonad	NA	-	LHCGR:NM_000233:c.C1573T:p.Q525*, Hom, parents	NA/ LOF	P/NA/P [Imen Et al 2015]
**DSD11**	19Y/F	25(6.3–24)	44(29.6–60)	NA	F	Absent uterus, vagina present, R ovary:18 mm/L ovary:26 mm	NA	Ovarian-like tissue	-	ZNRF3:NM_001206998:c.A1014G:p.I338M, Het, NA	Novel/ SIFT:D (0.01)/PP2:PD(0.519)/ REVEL:0.461(LB)	LP /NA/NA
**DSD12**	40D/F	NA	NA	NA	F	NA	NA	NA	Hydrocephalus,skeletal malformations,bilateral anophtalmos,agenesis of the corpus callosum	HHAT:NM_018194.6:c.C934A:p.R312S, Hom, parents	0.00006482 European/ SIFT:T(0.06)/PP2: PD(0.459) /REVEL:0.159(B)	LP/NA/NA
**DSD13**	3 Mo/M	NA	NA	NA	Micropenis, hypospadias, crytorchidism	NA	NA	NA	Dysmoprphic features,daibetes,epileptic seizures,atrophy of the supra tentorial level	IER3IP1: NM_016097.5:c.T62G:p.V21G,Hom, AR parents	0.0001491Latino admixed Americain /SIFT:D(0.00)/PP2:PD(0.583)/REVEL:0.886(LDC)	P/VUS/Khouloud et al. 2021]
**DSD14**	23Y/F	103.53(17–95	15.99(8–33)	NA	F	No ovaries, small uterus	No residual gonad	NA	NO	NSMF:exon3:c.134-4C > T, Het, NA	Novel/LOF	LB/NA/NA
										SEMA3A:c.G391A:p.A131T,Het, NA	European 0.0001176/ SIFT:T(0.16)/PP2:B(0.121) /REVEL:0.187(B)	B/NA/NA
**DSD15**	1Y/M	NA	NA	NA	Micropenis, hypospadias, small testes	NA	NA	NA	Dysmorphic features,psychomotor delay,hypotrophy	POR:NM_000941c.G1736A:p.R579Q, Het, NA	SIFT:T (0.13)/PP2:B(0.2)./REVEL:0.319(B)	B/NA/NA
										KDM3A:NM_001146688:c.C1535T:p.S512L, Het, NA	African American: 0.00004826/ SIFT:D(0.0)/PP2: PrD(0.98)/REVEL: 0.371(B)	LB/NA/NA
**DSD16**	16Y/F	48.33(6.3–24)	20.20(29.6–60)	NA	F	Infantile uterus, no ovaries	No residual gonad	NA	-	SOX9:NM_000346:c.C920G:p.P307R, Het, NA	European 0.00001470 / SIFT:T(0.05)/PP2:B(0.009)/REVEL: 0.426(LB)	LP/NA/NA
**DSD17**	2Y/M	NA	NA	NA	Left testicular ectopy	NA	NA	NA	Dysmorphic features/partial agenesis of the corpus callosum/microcephaly	PEX1:NM_000466:c.G2528A:p.G843D, Hom, parents	African American 0.00009653/ SIFT:D(0.0)/PP2: PrD(1.0)/REVEL: 0.984(LDC)	P/P/NA
**DSD18**	1Y and 1Mo/M	NA	NA	NA	Hypospadias,micropenis, unilateral cryptorchidism		R: in place L: in the abdomen	Testes/NA	Dysmorphic features,CIV,intra-uterine growth delay	POLE:NM_006231:c.C1707G:p.F569L,Het, parents	Latino/American 0.00006545 / SIFT:T(0.11)/PP2: B(0.04) /REVEL:0.171(B)	B/VUS/NA
										ANOS1:NM_000216:c.C1283T:p.P428L, Hemi, parents	european 0.00005634/ SIFT: T(0.73)/PP2:B(0.00)/REVEL:0.081(B)	LB/LB/NA
**DSD19**	4Mo/M	NA	NA	NA	Micropenis, cryptorchidism, small smooth scrotum	NA	NA		-	MAMLD1:NM_005491:c.A862C:p.M288L, Hemi, maternal	0.00005637 european/SIFT:T(0.17)/PP2:B(0.053)/REVEL:0.057(B)	B/NA/NA
**DSD20**	15D/M	NA	NA	NA	Anterior interscrotal hypospadias, micropenis, hypoplasic scrotum, left testicular ectopy	Bilateral hydrocele	R: no residual gonad, L:inguinal region	Testis/NA	-	FGF17:NM_001304478:c.C32T:pT11I, Het, NA	0.00001470 european/ SIFT:T(0.17)/ PP2:B(0.053)/ REVEL:0.256(B)	VUS/NA/NA
**DSD21**	36Y/M	116(1.5–10)	NA	0.52(2.5–10)	Small left testis	R: no testis L: atrophic testis		Testis/NA	-	NSMF:NM_015537:c.A125G:p.N42S, Het, NA	Novel/ SIFT:D(0.005)/PP2:B(0.417)/REVEL: 0.069(B)	B/NA/NA
**DSD22**	4Y and 9Mo/M	NA	NA	NA	Micropenis, small scrotum	Testis in the inguinal region	R:inguinal,L:inguinal	Testis/NA	Epilepsy,coortical atrophy	PDYN:NM_024411:c.34delC:p:L12fs, Het, NA	Novel	VUS/NA/NA
										MAMLD1:NM_001177465:c.C2573T:p.P858L, Het, maternal	European 0.0001506/SIFT:D/PP2:PD/REVEL: 0.188(B)	VUS/VUS/NA
**DSD23**	21Y/M	NA	NA	NA	Hypospadias, small testis	Testis in scrotum	NA	Testis	Hypothyroidism/low hairness	ARID1B:NM_001346813:c.1053_1054insGGC:p.G351delinsGG,Het, NA	Novel/LOF	VUS/VUS/NA
										, INSR:NM_000208:c.T3410C:p.I1137T, Het, NA	European 0.000008790/SIFT:D/PP2:PD/REVEL:NA	VUS/VUS/NA
										INSR:NM_000208:c.G3034A:p.V1012M, Het, NA	East Asian 0.0001930 SIFT:D(0.02)/PP2:PD(0.546)/REVEL: 0.634(LDC)	VUS/VUS/NA
**DSD 24**	4Y and 6Mo/M	NA	NA	NA	Hypospadias, cryptorchidism	NA	L & R: abdomen	Testis/NA	-	FLNA:NM_001110556:c.G1019T:p.R340L, Hemi, maternal	Africain/Americain 0.00008078/ SIFT:D(0.01)/PP2:PrD(0.966)/REVEL:0.538(LDC)	LP/VUS/NA
**DSD25**	15Y/F	NA	NA	NA	F	Immature uterus without uterine cavity, vagina	No residual gonad	NA	-	NBN:NM_002485.5:c.C798T: p.R215W, Het, NA	European 0.001985/ SIFT:D(0.00)/PP2:PrD(0.977)/REVEL: 0.343(B)	LB/VUS/NA
**DSD26**	1Y and half/M	NA	NA	NA	Micropenis, cryptorchidism	NA	NA	NA	Dysmorphic features	AMH:NM_000479:c.C553G:p.Q185E, Het, NA	European 0.0002059// SIFT: D(0.01)/PP2: PrD(0.925) /REVEL:0.492(B)	LB/LB/NA
**DSD27**	39Y/M	NA	NA	NA	Micropenis	No testes	NA	NA	-	POR:NM_000941:c.C344T:p.A115V, Het, NA	European 0.0001911/ SIFT:T(0.04) /PP2:B(0.408) /REVEL:0.481(LB)	LB/LB/NA
**DSD28**	1Y and half/M	NA	NA	NA	Unilateral crytorchidism	NA	R:abdomen L: scrotum	NA	Dysmorphic features,mental retardation,clinodactyly,	FRAS1:NM_025074:c.A7622G:p.N2541S, Hom, parents	European 0.0005442/ SIFT: D(0.03)/polyphen2: PrD(0.997) /REVEL:0.177(B)	B /LB/NA
**DSD29**	8Mo/M	NA	NA	NA	Hypospadias/cryptorchidism	Testes in inguinal region	L & R: inguinal region	Testes/NA	Dysmorphic features/hypotonia/microcephaly/fallot tetralogy/brachycaphaly	ANKRD11:NM_001256183:c.C5578T:p.P1860S, Het, NA	European 0.0005580/ SIFT: T(0.07)/PP2: LB(0.348) /REVEL:0.148(B)	LB/LB/NA
**DSD30**	21Y/F	32.6(3–15)	53.3(1.2–12.5)	NA	F	Absent vagina and uterus	No residual gonad	NA	-	SOX8:NM_014587:c.A676C:p.T226P, Het, NA	Novel/ SIFT:D(0.04)/PP2: ¨PrD(0.99)/REVEL:0.812(LDC)	LP/NA/NA
**DSD31⃰**	33Y/F	57 (3–12)	11.7 (1.1–7)	0.4 (3–12)	F	Bilateral gonadal agenesis	No residual gonad	Small impubertal uterus, Fallopian tubes	-	SRY:NM_003140.3c.C188A:p.P63H, Hemi, NA	Novel/ SIFT: D(0.00)/PP2: PrD(1.00)/REVEL: 0.720(LDC)	LP/NA/NA

Abbreviations: **ACMG** american college of medical genetics, **AD** autosomal dominant, **AMH** anti-Müllerian hormone, **AR** autosomal recessive, **B** benign, **D** day, **D** deleterious,** DSD** disorders/differences of sex development, **F** female, **FSH** follicle stimulating hormone, **gnomAD** genome aggregation database, **GV** gene variants previously associated with the disease, **Hemi** hemizygous, **Het** heterozygous, **Hom** homozygous, **L** left, **LB** likely benign, **LDC** likely disease causing, **LH** luteinizing hormone, **LOF** loss-of-function, **LP** likely pathogenic, **LPG** left palpable gonad, **M** male, **MAF** minor allele frequency, **MD** Mullerian ducts, **Mo** month, **NA** not available, **P** pathogenic, **PD** possibly damaging, **PP2** polyphen V2.2, **PrD** probably damaging, **R** right, **Ref** reference, **REVEL** rare exome variant ensemble learner, **Sibs** siblings, **VUS** variant of uncertain significance, **WES** Whole exome sequencing, **Y** year, * identified with direct sanger sequencing

The most common genetic diagnosis was variants in the androgen receptor (26%, 7/27).

**Fig. 2:**
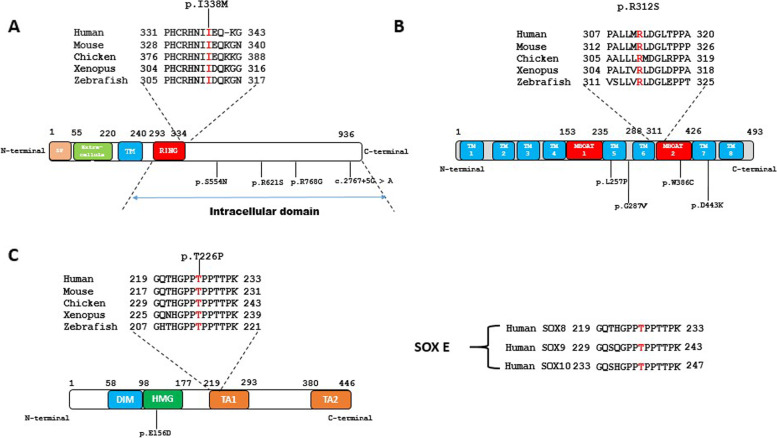
**A** Schematic representation of ZNRF3 protein indicating the known functional domains. The sequence alignment indicating the position and evolutionary conservation of the mutated isoleucine 338 residue, immediately adjacent to the RING finger domain. Previously published variants linked to 46,XY DSD are shown and located within the intracellular domain. **B** Schematic representation of HHAT protein indicating the position of the mutated p.R312 residue. Other published variants associated with this syndromic form of 46,XY DSD are indicated. **C** Representation of the SOX8 protein showing the position of the mutated p.T226 residue located in the evolutionary conserved TA1 domain. The only other SOX8 variant known to be associated with 46,XY DSD is the p.E156D mutation located within the HMG-box. Right, the mutated threonine residue is conserved in the SOXE group of proteins. DIM, DNA-dependent dimerization domain; HMG, high mobility group; MBOAT, Membrane Bound O-Acyltransferase domain; TA, transactivation domain; TM, transmembrane domain; SP, signal peptide

A *de novo* pathogenic variant (p.S426*) in the *AR* gene was observed in two sisters who presented complete androgen insensitivity syndrome (CAIS). Two other affected girls with CAIS from unrelated families (DSD3 and DSD4) shared a pathogenic variant (p.G744E), suggesting a possible founder effect. We identified novel or rare likely pathogenic variants in the *ZNRF3* (DSD 11), *HHAT* (DSD 12), and *SOX8* genes (DSD 30). A girl with 46,XY complete gonadal dysgenesis carried novel missense heterozygous *ZNRF3* variant (p.I338M). According to SIFT (0.01), PP2 (0.519) and REVEL (0.461) scores, this variant is likely to be disease causing. Isoleucine 338 is a highly conserved residue within the long intracellular domain (Fig. 2A), immediately adjacent to the ring domain (amino acids 293–334), which is responsible for the E3 ubiquitin ligase activity. A newborn 46,XY girl (DSD 12) presented hydrocephalus, skeletal malformations, bilateral anophtalmos and agenesis of the corpus callosum carried a very rare homozygous variant (p.R312S) in *HHAT* gene (DSD 12). The evolutionary conserved p.R312 residue is located in the Membrane Bound O-Acyltransferase domain 2 (MBOAT 2; Fig. 2B), which is required to palmitoylate Hedgehog proteins including SHH and DHH [16]. The *in silico* tools PP2 (0.99), SIFT (0.04) and REVEL (0.812) showed that this variant is likely to be disease causing. Hope tool predicted this variant to be damaging for the protein since the mutation introduces a more hydrophobic residue at this position and this can result in loss of hydrogen bonds and/or disturb correct folding. A novel heterozygotic p.T226P variant in *SOX8* gene was identified in a 46,XY female with probable testicular regression syndrome (high FSH, LH levels, no residual gonad, absent vagina and uterus). The T226 residue, located within the transactivation domain 1, is highly conserved among vertebrates and within the SOXE group of proteins (Fig. 2C). PP2 prediction tool indicated that this variant is likely disease causing. Hope predicted this variant to be likely damaging to the protein since it is located in an important domain for the main activity of the protein. The charge of the wild-type residue will be lost, and that change can cause loss of interactions with other molecules or residues. The inheritance pattern of both the *ZNRF3* and *SOX8* variants is unknown as parents DNA was unavailable. Both variants are absent from all public databases.

A rare homozygous variant in *PEX1* gene (p.G843D) was identified in a child boy (DSD 17) with syndromic form of DSD, including microcephaly, partial agenesis of the corpus callosum, dysmorphic features and unilateral cryptorchidism. SIFT (0), PP2 (1) and REVEL (0.984) prediction tools indicated that this variant is likely disease causing.

## Discussion

Sex chromosome as well as autosomal anomalies were present in 30.9% of the 46,XY DSD cohort, with the majority classified as 47,XXY Klinefelter’s syndrome. This is similar to frequencies reported by Mazen et al., 2021 studying a North African cohort, but higher than those reported in other studies [17]. As suggested by Mazen et al., 2021, this rate may be due to a recruitment bias as the research center in Tunisia is a reference centre for cytogenetics. However, it indicates that a considerable proportion of 46,XY DSD cases is due to chromosome anomalies that can be detected during routine karyotyping. aCGH detected further 8 individuals with chromosomal anomalies, associated with 46,XY DSD in 4 patients.

WES is considered the best method for identifying disease causing gene variants in DSD due to the complexity of the phenotypes [18]. Current data indicate that approximately more than half of patients with 46,XY DSD still lack a definite clinical diagnosis at the genetic level after WES [10, 19]. In this North African cohort of DSD, the genetic cause was established in 41.2% (68/165) of the total cohort, with a genetic cause identified in 38.6% of patients following WES. Recent cohort studies, using WES rather than targeted NGS panels have given a diagnosis yield in 46,XY DSD cohorts of 43% and 51% respectively [10, 20]. The lower yield of 38.6% reported here may reflect the proportion of undervirilised men in the cohort, a group that is difficult to reach a definitive clinical diagnosis or establish a genetic etiology [21, 22]. However, similarly to other studies the most common genetic cause was hemizygous variants in the *AR* [23, 24]. A total of 7 individuals, including two sisters, carried pathogenic variants in the AR. The G744E variant was observed in two unrelated patients, suggesting a possible founder effect for this variant.

A proportion of XY males carrying deletions of 8p23.1 that encompasses the *GATA4* gene have hypospadias and bilateral cryptorchidism [25, 26]. Here, a 46,XY female with atypical external genitalia (micropenis, small palpable right testis) carried a 4 Mb microdeletion in the 8p23.1 encompassing the *GATA4* gene [27, 28]. Pathogenic variants in *GATA4* have been identified in 46,XY DSD with or without cardiac heart defect [27–29]. To our knowledge this is the first case with a 8p23 microdeletion in a patient with 46,XY DSD raised as female.

WES revealed several very rare causes of 46,XY DSD including the genes *ZNRF3*, *SOX8* and *HHAT*. A novel heterozygous missense variant (p.I338M) in *ZNRF3* was identified in a 46,XY female with complete gonadal dysgenesis (DSD11). *ZNRF3* functions in testis-determination by inhibiting canonical pro-ovary WNT signaling pathway in XY gonads [30]. *ZNRF3* does this by targeting Frizzled receptors for degradation by ubiquitination and increased membrane turnover [31]. A total of four rare or novel heterozygous variants (3 missense and one splice region) in *ZNRF3* have been reported with both mild and severe 46,XY DSD [30]. All of these variants, including the p.I338M reported here, are located within the C-terminal intracellular domain portion of the protein [31], suggesting a possible genotype/phenotype correlation. SOX8 is an high mobility group (HMG)-box transcription factor, which is co-expressed with *SOX9* and *NR5A1/SF1* in testis-determination. *SOX8* shows functional redundancy with *SOX9* and may represses Foxl2 expression [32–34]. Heterozygous missense variants in *SOX8* are associated with either male or female infertility. Although rearrangements at the *SOX8* locus are associated with 46,XY gonadal dysgenesis, only a single pathogenic missense variant, located within the conserved HMG domain (p.E156D), has been demonstrated to cause 46,XY gonadal dysgenesis [35]. Here, a novel heterozygous missense variant p.T226P, located within transactivation (TA) domain, was carried by a 46,XY female with testicular regression syndrome. The p.T226 residue is conserved within the SOXE group of proteins, suggesting a functional role. The mode of inheritance of the *ZNRF3* and *SOX8* variants mutation is unknown, as the parents were unavailable for study. Hedgehog acyltransferase (HHAT) is an ER-resident multipass membrane protein consisting of 10 transmembrane domains and 2 re-entrant loops [36]. It is a member of the membrane bound-O-acyltransferase (MBOAT) family of enzymes that catalyze the attachment of specific fatty acids to secreted proteins [37]. *Hhat*^*−/−*^ mice display severely impaired development of fetal Leydig cells, Sertoli cells and testis cords[16]. In humans, biallelic pathogenic variants in *HHAT* are very rare and associated with a wide spectrum of neurodevelopmental phenotype including microcephaly, cerebellar vermis hypoplasia, gonadal dysgenesis, seizures and thinning of corpus callosum [16, 38, 39]. Only four families have been described in the literature and the common features are microcephaly and gonadal dysgenesis. Here, we identified a novel homozygous missense variant (p.R312S) in the conserved MBOAT domain-2 of *HHAT* carried by a 46,XY female with somatic anomalies including hydrocephalus, agenesis of the corpus callosum, skeletal malformations and bilateral anophtalmia.

## Conclusion

A combination of cytogenetics and exome sequencing can explain the genetic cause of 46,XY DSD in just over 40% of all cases. Exome sequencing is particularly useful in detecting very rare genetic causes of DSD in genes such as *ZNRF3, SOX8* or *HHAT* that would otherwise have been difficult to determine using other approaches.

## Data Availability

Please contact the author for data requests.

## Abbreviations

aCGH: Array comparative genomic hybridization
DNA: Desoxyribonucleic Acid
DSD: Differences/disorders of sex development
HMG: High mobility group
KS: Klinefelter syndrome
WES: Whole exome sequencing
